# Mechanism-Based Management of Pain in Chronic Pancreatitis: An Integrated Narrative Review

**DOI:** 10.7759/cureus.104097

**Published:** 2026-02-23

**Authors:** Saroj K Sahu, Bipadabhanjan Mallick, Chandramauli Mishra, Preetam Nath, Dibyalochan Praharaj, Suprabhat Giri, Anil C Anand, Manoj K Sahu

**Affiliations:** 1 Gastroenterology and Hepatology, Kalinga Institute of Medical Sciences, Bhubaneswar, IND

**Keywords:** abdominal pain, central sensitization, chronic pancreatitis, endoscopic therapy, eus-guided pancreaticogastrostomy, pain mechanisms, surgery

## Abstract

Pain is the most frequent and debilitating manifestation of chronic pancreatitis (CP), substantially impairing quality of life and driving healthcare utilization. Despite advances in imaging and interventional therapies, effective pain control remains challenging because pain mechanisms are heterogeneous and often poorly correlated with the extent of structural pancreatic disease. Contemporary evidence indicates that CP-related pain arises from interacting nociceptive, neuropathic, and nociplastic pathways, shaped by pancreatic ductal obstruction, ongoing inflammation, neural remodeling, and central sensitization. This narrative review summarizes and contextualizes current evidence on the epidemiology, pathophysiology, and management of pain in CP, with a focus on a mechanism-based, multidisciplinary treatment approach. Conservative management, including lifestyle modification, nutritional optimization, pancreatic enzyme replacement for exocrine insufficiency, and stepwise analgesic and neuromodulatory pharmacotherapy, forms the cornerstone of care. Endoscopic and endoscopic ultrasound-guided interventions are beneficial in carefully selected patients with large-duct obstruction or identifiable nociceptive drivers but have limited efficacy in small-duct or centrally mediated pain. Surgical intervention offers the most durable pain relief in appropriately selected patients, particularly when undertaken early, before the establishment of central sensitization or opioid dependence; randomized trials support early surgery over prolonged endotherapy in painful obstructive CP. Recurrent pain following surgery is multifactorial and necessitates systematic reassessment to distinguish remediable structural disease from neuropathic or nociplastic pain. Overall, aligning therapeutic strategies with pancreatic ductal morphology and dominant pain mechanisms is critical to achieving sustained pain relief, minimizing opioid use, and improving long-term outcomes.

## Introduction and background

Chronic pancreatitis (CP) is a progressive fibro-inflammatory disease characterized by irreversible structural damage to the pancreas, leading to exocrine and endocrine dysfunction in individuals with genetic susceptibility or environmental risk factors [1,2]. Abdominal pain is the most common and clinically relevant manifestation of CP and represents a major contributor to healthcare utilization and impaired quality of life (QoL). Pain in CP results from pancreatic inflammation, fibrosis, ductal hypertension, and perineural changes that activate and sensitize nociceptors, with progression to central sensitization and altered pain processing. Consequently, pain may become chronic and partially independent of structural pancreatic damage. Despite advances in understanding disease mechanisms and the availability of multiple therapeutic options, pain in CP remains challenging to manage, with considerable variability in clinical practice and outcomes.

This review aims to provide a comprehensive, clinically oriented overview of pain in CP by summarizing current concepts of pain pathophysiology and critically assessing available medical, endoscopic, and surgical management strategies. In particular, it seeks to address gaps in the existing literature by integrating emerging insights into pain mechanisms with pragmatic treatment algorithms, highlighting areas where evidence is limited or conflicting, and clarifying how available data complement, rather than duplicate, existing guidelines and prior reviews.

Epidemiology

Pain is reported in approximately 60-80% of patients during CP. Longitudinal data indicate that ~85% of patients develop persistent pain within four to five years of disease onset, while up to 60% continue to experience painful episodes beyond 10 years [3-5]. The prevalence of pain is particularly high in Asian populations, with Indian cohorts reporting rates exceeding 90%. The etiological distribution of CP varies by geographic region. Alcohol remains the predominant cause in Western countries, whereas idiopathic chronic pancreatitis (ICP) is the most common etiology in India, although alcohol-induced chronic pancreatitis (ACP) is increasingly recognized [6,7]. Pain is more frequently reported as a presenting symptom in ICP than in alcoholic CP. Over 90% of patients with both early- and late-onset ICP present with pain; however, pain onset occurs earlier in early-onset disease, whereas late-onset ICP is associated with higher rates of diabetes mellitus and exocrine pancreatic insufficiency [8].

Pain in CP is heterogeneous, manifesting as intermittent or persistent symptoms reflecting underlying pathophysiological differences [9]. European cohorts predominantly describe intermittent pain patterns, while persistent pain correlates with structural complications, increased healthcare utilization, disability, and impaired QoL, with active smoking being a consistent adverse prognostic factor [10,11]. Pain burnout is more likely in late-onset, non-calcific, idiopathic disease with intermittent pain and absence of ductal obstruction, particularly among non-smokers, and often parallels progression to exocrine pancreatic insufficiency or diabetes mellitus, reflecting glandular burnout [12,13].

## Review

Search methodology

A comprehensive narrative literature search was conducted to identify key evidence on the epidemiology, pathophysiology, and management of pain in CP. Major electronic databases, including PubMed/MEDLINE, Embase, Scopus, and the Cochrane Library, were searched from database inception to December 2025, using relevant Medical Subject Headings and free-text terms such as CP, pain, analgesic therapy, endoscopic interventions, celiac plexus block, and surgical management. Reference lists of pertinent review articles, consensus statements, and international clinical practice guidelines were manually screened to identify additional relevant publications. Studies were selected for inclusion based on clinical relevance and contribution to understanding pain mechanisms or informing management strategies, with an emphasis on randomized controlled trials, high-quality observational studies, systematic reviews, and guideline documents published in English. Given the narrative (non-systematic) nature of this review, formal inclusion-exclusion criteria and quantitative quality assessment of individual studies were not applied, and the search strategy is inherently subject to selection bias.

Pathophysiology of pain

**Figure 1:**
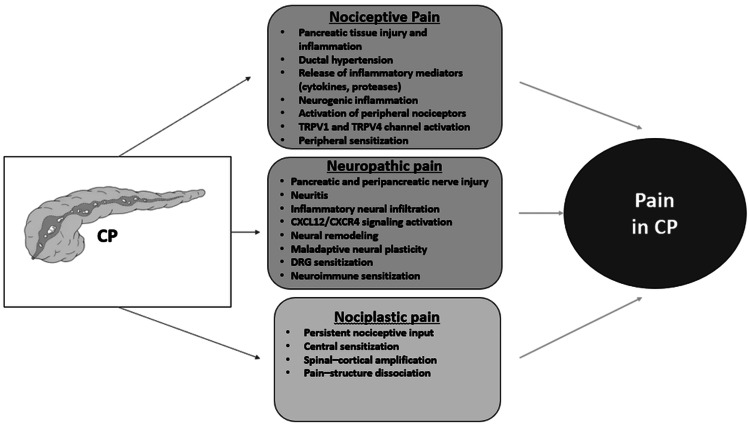
Mechanisms of pain generation in chronic pancreatitis CP, chronic pancreatitis; TRPV1, transient receptor potential vanilloid 1; TRPV4, transient receptor potential vanilloid 4; CXCL12, C-X-C motif chemokine ligand 12; CXCR4, C-X-C chemokine receptor 4; DRG, dorsal root ganglion; TRP, transient receptor potential Image credit: Dr. Saroj K. Sahu

Pain in CP is multifactorial, arising from pancreatic or extrapancreatic sources, including nociceptive, neuropathic, and nociplastic mechanisms that often overlap. This complexity frequently contributes to treatment failure (Figure 1) [14,15].

Nociceptive pain results from pancreatic tissue injury and inflammation, whereas neuropathic pain arises from damage to pancreatic or extrapancreatic nerves. Nociceptive pain in CP results from activation of nociceptors by tissue injury and inflammation, with peripheral sensitization lowering pain thresholds. This process is sustained by neurogenic inflammation, cytokines, proteases, and mediators that act on ion channels, such as transient receptor potential vanilloid (TRPV) 1 and 4, and voltage-gated A-type (IA) potassium currents (Figure 1). Other substances, like transforming growth factor-beta 1 (TGF-β1), platelet-derived growth factor B (PDGF-B), and glycoprotein 130 (GP130), are present; patients complain of dull, sharp, or nagging pain in the upper abdomen with radiation to the back [16,17].

Neuropathic pain in CP results from injury-induced modifications in pancreatic and peripancreatic nerve signaling. It is driven by neuroplasticity, neural remodeling, neuritis, inflammatory infiltration, and dorsal root ganglion (DRG)-mediated amplification of pain, with C-X-C motif chemokine ligand 12/C-X-C chemokine receptor type 4 (CXCL12/CXCR4) signaling further enhancing neuro-immune sensitization (Figure 1). The DRG amplifies pancreatic pain through maladaptive neuroplasticity and sustained nociceptive signaling. Upregulated CXCL12/CXCR4 enhances neuro-immune sensitization, making DRG pathways promising therapeutic targets [18,19]. Over time, pain may become independent of ongoing pancreatic injury, resembling a neuropathic pain state influenced by psychological and psychosocial factors, which explains the poor correlation between structural disease severity and pain intensity. Altered descending inhibitory pathways and enhanced facilitatory pathways further perpetuate chronic pain. Cross-organ sensitization may also account for overlapping functional gastrointestinal symptoms seen in many patients.

Nociplastic pain in CP arises from altered central pain processing rather than ongoing tissue injury or nerve damage. It is driven by central sensitization, impaired descending inhibitory pathways, and persistent amplification of pain signals within the spinal cord and brain. Central sensitization develops through sustained nociceptive input, leading to amplified pain processing and persistence of pain independent of ongoing tissue injury. This mechanism also explains the disproportionate severity of pain relative to structural pancreatic disease (Figure 1) [17,20]. Neuroplastic changes, including neural hypertrophy, altered neurotransmission, and viscerosomatic convergence, also contribute to persistent and widespread pain.

There are two distinct types of clinical manifestations of pain in CP. The "A-type pain," or intermittent pain, is characterized by discrete episodes of pain with pain-free periods in between. The "B-type pain" is described as persistent background pain with episodes of acute exacerbation. Studies have shown that intermittent pain responds more predictably to treatment than the latter type ("B-type pain") [21,22].

Hence, the pathophysiology of pain in CP reflects a dynamic interplay between peripheral pancreatic injury and progressive central nervous system reorganization. While early pain is driven by inflammation, fibrosis, ductal hypertension, and perineural changes that sensitize pancreatic nociceptors, persistent nociceptive input ultimately promotes central sensitization, impaired descending inhibitory control, and maladaptive brain pain processing. As a result, pain may assume a chronic, neuropathic-like phenotype that is increasingly dissociated from structural pancreatic abnormalities. This mechanistic evolution accounts for the poor correlation between imaging findings and pain severity and highlights the necessity for individualized, mechanism-based, multimodal therapeutic approaches [23,24].

Management

Goals of Treatment

The primary goals of therapy for pain in CP are to achieve durable pain relief, improve QoL, and restore functional capacity while minimizing treatment-related morbidity. Management is best conceptualized as a combination of non-interventional and interventional strategies guided by the dominant pain mechanism [2,4]. Non-interventional therapy focuses on attenuating pancreatic stimulation and inflammation through lifestyle modification, PERT, and analgesic and neuromodulatory agents to address inflammatory, neuropathic, and centrally sensitized pain. Interventional treatments target pancreatic ductal hypertension and local complications, with endoscopic or surgical ductal decompression reserved for patients with obstructive large-duct disease or refractory pain. Across both approaches, preservation of residual pancreatic exocrine and endocrine function, prevention of disease progression, and reduction of long-term opioid dependence remain central objectives, supported by a stepwise, mechanism-based treatment paradigm [25]. Accordingly, the goal of pain management in CP should extend beyond reduction in pain scores to include improved QoL, minimization of opioid dependence, and the sustained ability to engage in daily activities.

Non-interventional Therapy

Non-interventional therapy should be tried in all patients with CP-related pain because pain mechanisms are multifactorial and often include inflammatory, neuropathic, and centrally mediated components that may respond to medical management. Early conservative treatment can reduce symptoms, limit opioid use, and help identify patients unlikely to benefit from invasive ductal or surgical interventions.

Non-pharmacologic Measures

Core non-pharmacologic measures include strict alcohol abstinence and smoking cessation, consistently recommended because both exposures worsen the disease course and are associated with pain exacerbations. Structured nutrition support (small, frequent meals; individualized fat restriction when symptomatic; correction of vitamin and micronutrient deficiencies) and treatment of comorbid anxiety/depression and sleep disturbance are also important for reducing the pain burden and improving function (Table 1) [26,27]. Alcohol exacerbates pain in CP by perpetuating pancreatic inflammation, increasing ductal obstruction and intraductal pressure, and promoting neural hypertrophy and central sensitization. Active smoking independently accelerates fibrosis and perineural inflammation, worsens neuropathic pain, and reduces responsiveness to medical, endoscopic, and surgical therapies, making abstinence from both exposures essential for pain control. Current society guidance emphasizes a multidisciplinary approach that integrates lifestyle modification, nutrition, and stepwise analgesia before escalating to invasive therapies [28,29].

**Table 1:** Comprehensive conservative management strategies for pain in chronic pancreatitis PERT, pancreatic enzyme replacement therapy

Domain	Components and key considerations
Lifestyle and etiologic control	Complete abstinence from alcohol and smoking; nutritional optimization and maintenance of healthy body weight
Dietary and nutritional therapy	Small, frequent, low-fat meals; use of medium-chain triglycerides in patients with persistent fat intolerance; adequate protein and caloric intake; correction of micronutrient deficiencies, particularly fat-soluble vitamins (A, D, E, and K)
PERT	High-dose enteric-coated pancreatic enzyme preparations administered with meals, often combined with acid suppression using proton pump inhibitors or H2 receptor blockers, reduce cholecystokinin-mediated pancreatic stimulation and are more effective in early disease and small-duct chronic pancreatitis
Analgesic therapy (WHO stepwise approach)	Step 1: Paracetamol with or without nonsteroidal anti-inflammatory drugs (used cautiously); Step 2: Weak opioids such as tramadol; Step 3: Strong opioids for refractory pain, prescribed for the shortest duration possible; long-term opioid escalation should be avoided
Neuromodulators	Pregabalin or gabapentin, amitriptyline, and duloxetine; particularly useful for neuropathic or centrally mediated (nociplastic) pain, especially in small-duct chronic pancreatitis; target central sensitization and pain amplification
Antioxidant therapy	Combination formulations containing selenium, vitamins C and E, methionine, and beta-carotene; evidence is modest but may benefit oxidative stress-related pain, particularly in non-alcoholic and small-duct chronic pancreatitis
Psychological and behavioral interventions	Cognitive behavioral therapy, structured pain-coping strategies, and management of comorbid anxiety or depression play a key role in chronic pain modulation and reduction of opioid dependence

Pharmacologic Measures

Pharmacologic conservative therapy generally follows a step-up strategy (often aligned pragmatically with the WHO analgesic ladder), prioritizing acetaminophen (when appropriate) and adjuvant agents over long-term opioids. Analgesic therapy should use oral, around-the-clock dosing guided by pain severity and drug pharmacokinetics, with individualized dose adjustment and strict adherence to balance efficacy and side effects and prevent pain recurrence [30]. Because a large component of CP pain is neuropathic/centrally mediated, neuromodulators (gabapentin/pregabalin, tricyclic antidepressants, and serotonin and norepinephrine reuptake inhibitors) are commonly used; notably, pregabalin demonstrated superiority to placebo for pain reduction in a randomized controlled trial and remains a key evidence-based option for selected patients [31]. If opioids are required, many experts recommend using the lowest effective dose, reassessing frequently, and integrating non-opioid adjuvants to minimize opioid exposure.

Most patients with CP do not achieve adequate pain control with non-opioid analgesics, leading to high reliance on advanced therapies; approximately three-quarters require opioid medications, nearly two-thirds undergo endoscopic interventions, and about one-third ultimately require surgical management. Optimal pain control requires a mechanism-based approach: nociceptive pain is best addressed with stepwise analgesia, lifestyle modification, and pancreatic ductal decompression via endoscopic or surgical methods; neuropathic pain responds more favorably to neuromodulators such as gabapentinoids and targeted interventions like celiac plexus blockade; while nociplastic pain benefits from multimodal strategies including structured physical activity, cognitive-behavioral therapy, mind-body interventions, antidepressants (e.g., duloxetine or tricyclics), acupuncture, and emerging neuromodulation techniques [15].

Adjunctive Therapies

Adjunctive therapies may be considered on a case-by-case basis. Pancreatic enzyme replacement therapy (PERT) is the standard treatment for pancreatic exocrine insufficiency (PEI) in CP and provides symptomatic relief by improving digestion and nutrient absorption. While it effectively reduces steatorrhea, diarrhea, and meal-related discomfort, its role in direct pain control is limited and inconsistent. PERT does not modify underlying inflammation or fibrosis and is therefore used as an adjunct to comprehensive pain management and lifestyle interventions to improve QoL [32]. Evidence does not support routine PERT solely for pain relief (particularly with enteric-coated preparations), so its role in analgesia is limited to selected scenarios and comorbidity management. Antioxidants have shown mixed results across trials and meta-analyses (including Cochrane data) and are best considered an optional adjunct in carefully selected patients rather than routine care [33]. Throughout conservative management, clinicians should reassess the pain phenotype, nutritional status, and psychosocial contributors, and refer for endoscopic or surgical evaluation only when a clear anatomic driver (e.g., obstructed main pancreatic duct (MPD)) is identified or when conservative measures fail. In large-duct CP, pain is predominantly driven by ductal obstruction and intraductal hypertension; conservative therapy provides supportive relief but often serves as a bridge to endoscopic or surgical ductal decompression. In small-duct CP, pain is largely nociplastic or neuropathic in origin; conservative management with enzymes, neuromodulators, antioxidants, and psychosocial interventions remains the cornerstone of long-term pain control [27,34].

Interventional management

ERCP-Guided Management

**Figure 2:**
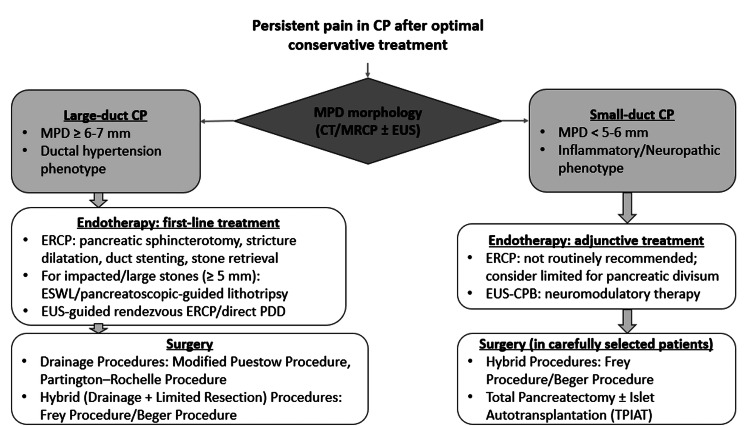
Algorithm for the management of persistent pain in chronic pancreatitis after failure of conservative therapy CP, chronic pancreatitis; MPD, main pancreatic duct; CT, computed tomography; MRCP, magnetic resonance cholangiopancreatography; EUS, endoscopic ultrasound; ERCP, endoscopic retrograde cholangiopancreatography; ESWL, extracorporeal shock wave lithotripsy; PDD, pancreatic duct drainage; EUS-CPB, endoscopic ultrasound-guided celiac plexus block; TPIAT, total pancreatectomy with islet autotransplantation Image credit: Dr. Saroj K. Sahu

Pain due to ductal obstruction with hypertension is the predominant nociceptive mechanism in patients with the large-duct phenotype, thus requiring ductal decompression (Table 2). Endoscopic retrograde cholangiopancreatography (ERCP)-guided ductal decompression is recommended as the first-line therapy for obstructive CP with ductal dilation due to stones or strictures, with optimal outcomes achieved through complete and sustained ductal decompression, particularly in large-duct disease (MPD ≥5-6 mm) (Figure 2). Conversely, ERCP is ineffective and not recommended in small-duct or non-obstructive disease, where pain is more likely to be neuropathic or centrally mediated. Across multiple studies, ERCP achieves technical success rates of approximately 70-90%, while clinical success, defined as meaningful pain relief, varies according to the underlying ductal pathology. Pain improvement is reported in 65-80% of patients with isolated ductal stones, 60-75% of those with dominant strictures, and decreases to <50-60% in patients with multiple strictures or combined complex disease. Importantly, durable pain relief correlates strongly with complete ductal clearance and sustained ductal decompression, rather than partial or transient interventions.

**Table 2:** Endotherapies for pain management in large-duct and small-duct chronic pancreatitis FCSEMS, fully covered self-expandable metal stent; ESWL, extracorporeal shock wave lithotripsy; ERCP, endoscopic retrograde cholangiopancreatography; EUS, endoscopic ultrasound

Characteristics	Large-duct chronic pancreatitis	Small-duct chronic pancreatitis
Pancreatic duct morphology	Dilated main pancreatic duct (≥5-6 mm)	Non-dilated main pancreatic duct (<5-6 mm)
Predominant pain mechanism	Ductal hypertension due to stones and/or strictures	Inflammatory and neuropathic pain with central sensitization
Primary therapeutic objective	Pancreatic duct decompression	Neural modulation and pain pathway interruption
ERCP-based interventions	Pancreatic sphincterotomy; ductal stone extraction; stricture dilatation; pancreatic duct stenting (plastic stents; FCSEMS in selected cases)	Limited role; generally ineffective unless a focal obstructive lesion is present
ESWL	First-line therapy for large (>5 mm), impacted pancreatic duct stones, followed by ERCP	No established role
Pancreatoscopy-directed lithotripsy	Electrohydraulic or laser lithotripsy for ESWL-refractory or radiolucent stones	Not indicated
EUS-guided ductal interventions	EUS-guided rendezvous pancreatic duct drainage or direct pancreatic duct drainage when ERCP fails	Not routinely indicated
EUS-guided neural interventions	Adjunctive role	EUS-guided celiac plexus block or neurolysis
Special indications	-	Minor papilla sphincterotomy with or without stenting in pancreas divisum
Expected pain relief	Higher likelihood of sustained pain relief after complete ductal clearance	Variable and often transient pain relief

In patients with large or impacted pancreatic duct stones (>5 mm), especially in the head or body, extracorporeal shock wave lithotripsy (ESWL) facilitates stone fragmentation and subsequent endoscopic extraction. Staged ERCP within 24-72 hours is preferred for optimal clearance. Pancreatoscopy-directed intracorporeal lithotripsy is reserved for radiolucent, impacted, or ESWL-refractory stones, preferably using laser lithotripsy, and should be considered a salvage option in expert centers [1,3,35].

ERCP is most effective for single, dominant strictures in the pancreatic head or proximal body with upstream ductal dilation. Long-segment, multiple strictures or those associated with an inflammatory head mass respond poorly to endotherapy, and repeated ERCP in these settings often delays definitive management without durable pain relief [36].

ERCP-based therapy should be discontinued when pain persists despite adequate ductal decompression, when stones or strictures recur rapidly despite optimized therapy, or when endoscopic treatment extends beyond 6-12 months without sustained clinical benefit. Temporary pancreatic duct stenting may provide short-term symptom relief or serve as a bridge to ESWL or surgery in patients with incomplete stone clearance; however, long-term stenting without definitive ductal clearance is not disease-modifying and should not be pursued as a stand-alone strategy. Several factors have been identified as predictors of futility of ERCP for pain control, including small-duct disease, multiple or diffuse strictures, extensive parenchymal fibrosis, long disease duration, opioid dependence, and features of central sensitization. Early recognition of these predictors is critical to avoid repeated ineffective endoscopic interventions and to facilitate timely referral for alternative therapeutic strategies [37].

Collectively, current evidence supports a step-up, mechanism-based approach to pain management in CP, in which ERCP remains the first-line interventional therapy for obstructive disease, supplemented by ESWL and advanced endoscopic techniques when indicated, withEndoscopic ultrasound (EUS)-guided interventions and surgery reserved for carefully selected patients who fail conventional endotherapy.

EUS-Guided Interventions

EUS-guided therapeutic interventions play an essential role in the management of pain in CP, particularly in patients with persistent symptoms despite optimized medical therapy and who are not immediate candidates for surgery (Figure 2). These are classified as neural (celiac plexus block or neurolysis) or pancreas-directed approaches, including EUS-guided pancreatic duct drainage (EUS-PPD) and rendezvous techniques [2,30].

Neural (Analgesic) EUS-Guided Interventions

Neural interventions include EUS-guided celiac plexus block (EUS-CPB) and EUS-guided celiac plexus neurolysis (EUS-CPN). EUS-CPB involves the injection of a local anesthetic with or without a corticosteroid around the celiac plexus or ganglia under real-time ultrasound guidance. It is indicated for patients with refractory pain despite ductal decompression or surgery, those without significant ductal obstruction, or early disease dominated by inflammatory neural sensitization (Figure 2) [35,38,39].

Performed under sedation, EUS-CPB uses anatomical landmarks including the aorta, celiac axis, and superior mesenteric artery, with injection of bupivacaine followed by triamcinolone using central or bilateral techniques. The procedure is generally safe, with mostly mild, transient adverse effects, and provides superior short-term pain relief compared with CT-guided approaches, although benefits are typically temporary.

Clinical response is variable and usually lasts weeks to months, supporting EUS-CPB as a palliative or adjunctive therapy rather than a definitive treatment. Better outcomes are seen in patients with visceral pain, shorter disease duration, and lower opioid requirements, while those with central sensitization respond poorly. Repeated blocks may be considered in responders, but failure after one or two sessions should prompt consideration of definitive surgical management.

Pancreas-Directed EUS-Guided Interventions

Advanced EUS-guided pancreatic duct procedures, like EUS-guided rendezvous techniques and direct EUS-PDD, are options for certain patients with blocked ducts who have not had success with ERCP (Figure 2). These procedures help relieve pressure in direct transmural drainage (pancreaticogastrostomy or pancreaticoduodenostomy), which is technically demanding and should be limited to high-volume expert centers.

EUS-guided rendezvous drainage involves EUS puncture of the pancreatic duct with guidewire passage across the papilla to enable transpapillary ERCP drainage, restoring physiological flow while preserving ductal anatomy and avoiding permanent transmural fistulae [40]. EUS-PDD entails duct puncture, pancreatography, tract dilation, and transmural stenting; most experts recommend an MPD ≥5 mm for safety and efficacy. Plastic stents (5-7 Fr) are standard, with fully covered self-expandable metal stents (FCSEMS) or lumen-apposing metal stents (LAMS) reserved for refractory cases in expert centers.

Technical success rates range from 82% to 95%, with clinical pain improvement in 80% to 93% of successful cases, particularly in patients with dilated ducts, clear obstruction, and limited central sensitization. Adverse events occur in 15-20% (pancreatitis, leak, infection, bleeding, perforation), reinforcing the need for careful selection and expert performance. These techniques are best considered salvage, anatomy-preserving options within a stepwise, mechanism-based approach, with surgery reserved for recurrent stent dysfunction or persistent symptoms [41,42].

Complication-Directed EUS-Guided Interventions

EUS-guided drainage of pancreatic pseudocysts and walled-off collections has been shown to significantly reduce pain when symptoms are driven by mass effect, ductal obstruction, or ongoing inflammation, particularly in patients who are poor surgical candidates. Significantly, the effectiveness of EUS-guided therapeutic interventions for pain in CP depends heavily on proper patient selection and a clear understanding of the underlying pain mechanisms, as outcomes are superior when nociceptive drivers are addressed rather than when pain is predominantly neuropathic or centrally mediated. Consequently, EUS-guided therapies should be viewed as integral components of a multidisciplinary, mechanism-based approach to refractory pain management in CP rather than as stand-alone treatments [2,35].

Surgery

Surgical management of CP includes decompression, resection, or combined procedures, selected according to pancreatic pathology. Although optimal timing remains debated, surgery should ideally be performed within two to three years of disease onset and before central sensitization develops. A step-up approach is recommended for large-duct disease, whereas small-duct disease warrants early surgical consideration with neuromodulatory strategies (Figure 2).

Large-Duct Disease

Surgery is indicated for persistent pain refractory to medical and endoscopic therapy, a markedly dilated MPD (≥7 mm), an inflammatory head mass, complications (biliary/duodenal obstruction), or suspected malignancy. Longitudinal pancreaticojejunostomy (Partington-Rochelle) is preferred for uniformly dilated ducts without a head mass. Head-dominant disease is best managed with combined drainage-resection procedures (Frey or Beger), which provide effective pain control with lower morbidity and better preservation of pancreatic function than pancreaticoduodenectomy. Pancreaticoduodenectomy is reserved for suspected malignancy or extensive head disease. Early surgery offers superior and more durable pain relief and quality-of-life benefits compared with endotherapy [42-44].

Small-Duct Disease

In patients with MPD <5-6 mm, pain is typically non-obstructive and unresponsive to endoscopic drainage. Early referral for surgery is recommended in refractory cases, particularly in young patients with preserved function or opioid dependence. Total pancreatectomy with islet autotransplantation (TPIAT) is the preferred option, especially in idiopathic or genetic disease, with the best outcomes when performed before central sensitization. Drainage procedures are ineffective, and resection has a limited role unless head-dominant disease is present [2,45].

Early Surgery Versus Endotherapy for Pain Control

Randomized evidence strongly favors early surgery over an endoscopy-first approach for sustained pain control in painful obstructive CP. In the ESCAPE trial, early surgery produced a significantly lower mean Izbicki pain score over 18 months than step-up endotherapy (37 vs 49; p=0.02), with patients undergoing fewer interventions (median one vs three procedures; p<0.001) [42]. At long-term follow-up (~eight years), complete pain relief was achieved in 45% of patients assigned to early surgery, compared with 20% in the endoscopy-first group (p=0.01), with persistently lower pain scores (33 vs 51; p=0.003) and higher patient satisfaction. Importantly, patients who crossed over from failed endotherapy to delayed surgery had significantly worse pain outcomes than those treated surgically upfront. These data support early surgical intervention, particularly in patients with a dilated MPD, complex ductal disease, or escalating opioid requirements, while endotherapy should be reserved for carefully selected, uncomplicated cases [43].

Management of Recurrent Pain After Surgery

Recurrent pain after surgery for CP is multifactorial, arising from persistent or recurrent structural disease (such as inadequate ductal decompression, anastomotic strictures, residual stones, inflammatory head mass, pseudocysts, or disconnected duct syndrome) as well as from neuropathic and nociplastic mechanisms related to neural inflammation and central sensitization [46,47]. Management requires a structured, stepwise, multidisciplinary approach, beginning with reassessment using cross-sectional imaging and/or EUS to identify potentially correctable pathology. Endoscopic therapy is preferred as first-line reintervention when treatable ductal abnormalities are present, and revisional surgery can effectively relieve symptoms in selected patients with persistent obstructive disease or failure of endotherapy. Optimized conservative management, including PERT, strict abstinence from alcohol and smoking, neuropathic pain modulators, nutritional support, and psychosocial interventions, remains essential in all patients irrespective of prior surgical intervention [47-49].

Limitations

Despite substantial advances in understanding the mechanisms and management of pain in CP, several important limitations persist. Much of the available evidence is derived from heterogeneous observational studies, small randomized trials, or expert consensus, resulting in variability in definitions of pain phenotypes, treatment endpoints, and outcome measures. Pain in CP is inherently subjective and multidimensional, yet standardized tools integrating nociceptive, neuropathic, and centrally sensitized pain components are not routinely applied in clinical practice, limiting comparability across studies. Additionally, most interventional trials focus on short- to medium-term pain relief, with limited long-term follow-up assessing the durability of the benefit, progression of pancreatic insufficiency, or opioid dependence. The relative lack of high-quality comparative trials evaluating endoscopic versus surgical strategies across clearly defined ductal and pain phenotypes further constrains evidence-based decision-making. In addition, many of the cited studies and guidelines originate from high-resource regions with specialized expertise, and the marked regional variability in access to advanced endoscopic, surgical, and multidisciplinary pain interventions further limits the generalizability of these recommendations to low-resource settings.

Future directions

In the management of pain in CP, there is an increasingly evident shift from a uniform, stepwise analgesic strategy toward a personalized, mechanism-based approach. Contemporary evidence underscores the heterogeneity of pain pathways in CP, encompassing ductal hypertension, ongoing inflammation, neuropathic pain, and central sensitization, which explains the frequent discordance between structural disease severity and pain outcomes. Advances in pain phenotyping, through clinical pain patterns, patient-reported outcomes, and quantitative sensory testing, offer a means to better identify centrally mediated pain states and optimize patient selection for endoscopic, surgical, or neuromodulatory therapies. Parallel progress in translational pain research and neuroimaging has enhanced understanding of peripheral and central nociceptive processing, creating opportunities for targeted pharmacologic interventions and opioid-sparing strategies. Emerging evidence underscores the value of multidisciplinary care models that integrate psychological and behavioral therapies to address the biopsychosocial nature of chronic pain. Accordingly, current guidelines and reviews call for well-designed randomized trials to validate personalized treatment algorithms, identify pain-related biomarkers, and define the optimal timing of invasive interventions, with the ultimate aim of achieving durable pain relief, improving QoL, and minimizing treatment-related morbidity in CP.

## Conclusions

Pain in CP represents a complex clinical challenge driven by overlapping nociceptive, neuropathic, and nociplastic mechanisms that are frequently discordant with structural disease severity. Contemporary evidence supports a stepwise, mechanism-based, and multidisciplinary approach to pain management, aligning therapeutic strategies with pancreatic ductal morphology and dominant pain pathways. Conservative medical therapy remains foundational, while endoscopic, EUS-guided, and surgical interventions should be selectively employed based on clear anatomic targets and the likelihood of durable benefit. Early identification of patients unlikely to respond to repeated endotherapy and timely referral for definitive surgical management are critical to preventing central sensitization and long-term opioid dependence. Future advances in pain phenotyping, biomarkers, and personalized treatment algorithms are expected to further improve long-term pain control and QoL in patients with CP.
